# Preliminary protocol for measuring the reproducibility and accuracy of flow values on digital PET/CT systems in [^15^O]H_2_O myocardial perfusion imaging using a flow phantom

**DOI:** 10.1186/s40658-024-00654-y

**Published:** 2024-07-01

**Authors:** Reetta Siekkinen, Heidi Partanen, Linda Kukola, Tuula Tolvanen, Andrew Fenwick, Nadia A. S. Smith, Mika Teräs, Antti Saraste, Jarmo Teuho

**Affiliations:** 1 Turku PET Centre, University of Turku, Turku, Finland; 2 Turku PET Centre, Turku University Hospital and Wellbeing Services County of Southwest Finland, Turku, Finland; 3 Department of Medical Physics, Turku University Hospital and Wellbeing Services County of Southwest Finland and University of Turku, Turku, Finland; 4 National Physical Laboratory, Teddington, UK; 5 Department of Physics and Astronomy, University of Turku, Turku, Finland; 6 Institute of Biomedicine, University of Turku, Turku, Finland; 7 Heart Centre, Turku University Hospital and Wellbeing Services County of Southwest Finland and University of Turku, Turku, Finland

**Keywords:** PET/CT, Myocardial perfusion imaging, Flow phantom, Test-retest reproducibility, Technical factors, Radiowater

## Abstract

**Background:**

Several factors may decrease the accuracy of quantitative PET myocardial perfusion imaging (MPI). It is therefore essential to ensure that myocardial blood flow (MBF) values are reproducible and accurate, and to design systematic protocols to achieve this. Until now, no systematic phantom protocols have been available to assess the technical factors affecting measurement accuracy and reproducibility in MPI.

**Materials and methods:**

We implemented a standard measurement protocol, which applies a flow phantom in order to compare image-derived flow values with respect to a ground truth flow value with [^15^O]H_2_O MPI performed on both a Discovery MI (DMI-20, GE Healthcare) and a Biograph Vision 600 (Vision-600, Siemens Healthineers) system. Both systems have automatic [^15^O]H_2_O radio water generators (Hidex Oy) individually installed, allowing us to also study the differences occurring due to two different bolus delivery systems. To investigate the technical factors contributing to the modelled flow values, we extracted the [^15^O]H_2_O bolus profiles, the flow values from the kinetic modeling (Qin and Qout), and finally calculated their differences between test-retest measurements on both systems.

**Results:**

The measurements performed on the DMI-20 system produced Qin and Qout values corresponging to each other as well as to the reference flow value across all test-retest measurements. The repeatability differences on DMI-20 were 2.1% ± 2.6% and 3.3% ± 4.1% for *Qin* and *Qout*, respectively. On Vision-600 they were 10% ± 8.4% and 11% ± 10% for *Qin* and *Qout*, respectively. The measurements performed on the Vision-600 system showed more variation between Qin and Qout values across test-retest measurements and exceeded 15% difference in 7/24 of the measurements.

**Conclusions:**

A preliminary protocol for measuring the accuracy and reproducibility of flow values in [^15^O]H_2_O MPI between digital PET/CT systems was assessed. The test-retest reproducibility falls below 15% in majority of the measurements conducted between two individual injector systems and two digital PET/CT systems. This study highlights the importance of implementing a standardized bolus injection and delivery protocol and importance of assessing technical factors affecting flow value reproducibility, which should be carefully investigated in a multi-center setting.

**Supplementary Information:**

The online version contains supplementary material available at 10.1186/s40658-024-00654-y.

## Introduction

Several factors may decrease the accuracy of Positron Emission Tomography (PET) quantification within one system or between different PET systems. These factors involve injection protocols, practical data acquisition with different kind of clinical protocols, different PET detector technologies, software implementations as well as reconstruction and data corrections [1–3]. The contribution of these factors is an additional challenge in myocardial perfusion imaging (MPI) quantification, as it relies on kinetic modelling to extract the myocardial blood flow (MBF) in absolute quantitative values, requiring accurate and reproducible image quantification.

The European Association of Nuclear Medicine Research Ltd. (EARL) has proposed guidelines for PET acquisition harmonization in order to achieve reproducible [^18^F]FDG tumor standardized uptake value (SUV) quantification [4, 5]. Similar procedural guidelines have been proposed for MPI, which frequently uses short lived tracers, such as [^15^O]H_2_O [6]. Implementing harmonization measures for MPI is extremely important. Any test used for clinical decision-making and patient management needs to be accurate and should have adequate test–retest repeatability to enable management decisions for individual patients [2, 7]. As MPI PET is increasingly being used for the detection of myocardial ischemia [6], there is a growing need for more consistent and standardized evaluation of the technical factors contributing to the variation of MBF values in MPI studies [3].

The lack of suitable test objects has hindered the implementation of standardized protocols for harmonization of MPI. Phantoms used in PET harmonization studies are usually static phantoms, such as the National Electrical Manufacturers Association (NEMA) image quality phantom [8] or simple cylindrical phantoms [9]. Few suitable phantoms have been available for harmonization purposes in dynamic MPI until recently. Gabrani-Juma et al. validated a flow phantom simulating MBF for MPI PET standardization purposes where image-derived flow values can be evaluated against a ground truth reference value [10]. This flow phantom offers an optimal basis for creating standardized protocols for assessing the accuracy and reproducibility of MBF values across PET systems as well as for investigating the contribution of purely technical factors on kinetic modelling and MBF quantitation.

Several studies have investigated harmonization measures of accurate SUV quantification for oncological [^18^F]FDG and neurological studies. Akamatsu et al. list several investigations of the latest PET harmonization strategies [11]. For example, SUV harmonization can be based on the image uniformity and spatial resolution with tracer-specific phantoms [12]. EARL has also performed extensive work for evaluating reproducible SUV quantitation and accreditation, and the investigations include harmonization protocols with standard acquisition parameters [5]. Furthermore, SUV (more specifically, SUVmean, SUVmax, and SUVpeak) has been reported to have 10% coefficient of variation [13] and 27% reproducibility rate for SUVmean and SUVpeak, and 33% for SUVmax [14].

Multiple test-retest studies have also evaluated the reproducibility of MPI PET with [^13^N]NH_3_ as well as [^82^Rb] [15–17]. In addition, in [^15^O]H_2_O MPI PET the repeatability for stress MBF values has been reported to be 27% [18] and 25% [19]. However, to the best of our knowledge, no studies have systematically investigated the contribution of technical factors on the test-retest reproducibility and accuracy with a calibrated standard. Therefore, the first step for implementing systematic harmonization protocols should be to evaluate these technical factors contributing to both the accuracy and reproducibility of MBF values between different bolus injector and PET/CT systems against physical reference standards. These evaluations might eventually result in standard clinical acquisition protocols that are capable of producing similar quantitative values across different PET systems and reducing the variation due to technical factors in MPI.

In this study, we evaluate the different technical factors affecting the accuracy and reproducibility of MBF values using a preliminary measurement protocol with two [^15^O]H_2_O bolus injector systems of the same manufacturer, as well as two digital silicon photomultiplier (SiPM) -based PET/Computed Tomography (CT) systems of different vendors, using a flow phantom capable of simulating MBF.

## Materials and methods

### PET systems

The study was conducted in a single-centre setting (Turku PET Centre). The protocol was implemented on two PET/CTs in Turku PET Centre; Discovery MI with 20 cm axial field-of-view (FOV) (DMI-20, GE Healthcare, Milwaukee, US) and Biograph Vision 600 (Vision-600, Siemens Healthineers, Erlangen, Germany). The system performance parameters are presented in Table 1.

**Table 1:** Performance characteristics of DMI-20 and Vision-600

	Unit	DMI-20	Vision 600
**Number of Detector Rings**	-	4	8
**Transaxial FOV**	cm	70	78
**Axial FOV**	cm	20	26.1
**Crystal Material**	-	LYSO	LSO
**Crystal Array**	-	4 × 9	5 × 5
**Crystal Size**	mm × mm × mm	3.95 × 5.30 × 25.0	3.2 × 3.2 × 20.0
**Detector Type**	-	SiPM	SiPM
**Detector Array**	-	3 × 6	-
**Detector Active Area**	mm × mm	4 × 6	16 × 16
**Number of Output Channels**	-	-	16
**SiPM Pixel Array**	-	2 × 3	-
**Sensitivity**	cps/kBq	13.7	16.4
**Spatial Resolution**	rad @ 1 cm	4.1	3.5
**Peak NECR**	kcps	193	306
**Peak NECR Activity**	kBq/ml	21.9	32
**Peak NECR Scatter Fraction**	%	40.6	38.7
**Timing Resolution**	ps	375	210
**Energy Resolution**	%	9.4	-

### Flow phantom

The flow phantom used in this study is presented in detail in the paper of Gabrani-Juma et al. [10], to which an interested reader is directed for a more extensive description. In short, the flow phantom comprises a closed-circulation system, which pumps water from an exterior container into the circulation using a peristaltic pump. The system is located inside a plastic shell filled with water, shaped as the NEMA image quality phantom that simulates body attenuation and scatter.

The pump flow rate, named *Qpump* may be adjusted by the user to produce a desired range of flow values. An injection port is located after the peristaltic pump, from which the activity can be administered directly to the system. Thereafter, the injected activity propagates directly to an input chamber (with a volume of 15.7 ml) which simulates the left ventricle blood pool. The input chamber is connected to an exchange cylinder that contains a perforated tube from which the tracer permeates to the exchange cylinder. The exchange cylinder simulates the myocardium, and the water flow from the perforated tube into the exterior volume of the exchange cylinder simulates the blood perfusion. The volume of the exchange cylinder (*Vcyl*) is 160 ml.

*Qcyl* marks the flow rate coming out of the exchange cylinder whereas *Qtube* marks the flow rate coming out of the perforated tube. This is because not all water passes from perforated tube to the exterior volume of the exchange cylinder. There is a flow controller valve for both *Qcyl* and *Qtube* that allow the user to create different types of restricted and unrestricted flow inside the flow phantom. Qcyl and Qtube are measured using calibrated flow meters (Omega Engineering Inc., Norwalk, US). The reference flow value *Qref* is derived from *Qcyl* using the flow meter calibration factors and a look-up table.

The input and tissue time-activity curves (TACs) needed for the kinetic modelling can be measured from the input chamber and exchange cylinder, respectively. This analysis is performed semi-automatically using a software provided by the phantom vendor, where the regions of interest can be defined and then used to derive the TACs.

A phantom-specific two-compartmental (one-tissue) kinetic model is used, which is extensively explained in Gabrani-Juma et al. (Equations 2–6) [10]. The model includes two rate parameters, *qin* and *qout*, as well as an input signal fraction (*ISF*) and a *delay* parameter. *qin* [min^− 1^] denotes the rate parameter that describes the flow passing from the perforated tube into the exterior volume of the exchange cylinder, and *qout* [min^− 1^] the rate parameter that describes the flow coming out from the exchange cylinder. *ISF* [dimensionless] accounts for the exchange cylinder spill-over from the perforated tube and *delay* [s] defines the tracer passage time from the input cylinder to the perforated tube. *qin* and *qout* are analogous to *K1* and *k2*, the rate constants used in a two-compartmental kinetic model and commonly used to derive MBF e.g. for [^15^O]H_2_O [20, 21]. *qin* and *qout* are multiplied by *Vcyl* in order convert them to flow values *Qin* and *Qout* in [ml/min]. In the phantom, for an ideal measurement, *Qin* = *Qout* = *Qref*.

### Measurement protocol

The measurement protocol assessed 12 different flow values, from low to high. *Qpump* values were set to 150 ml/min, 200 ml/min, and 250 ml/min. *Qcyl* values were adjusted to 20%, 40%, 60%, and 80% of *Qpump* using the constrictor valves, to simulate reduced perfusion. *Qcyl* and *Qtube* values were recorded before and after the measurement and their mean values were applied as the reference flow values. For Vision test 150 − 20%, 150 − 40%, and 150 − 80% measurements the recorded *Qcyl* and *Qtube* were recorded before the measurement. Table 2 presents all recorded flow values from the phantom measurements.

**Table 2:** Acquisition parameters for all measurements. Qcyl marks the flow rate coming out of the exchange cylinder and Qtube marks the flow rate passing out of the perforated tube to the exchange cylinder

Measurement	Qpump	Constriction	Qcyl	Qcyl measured				Qtube	Qtube measured			
				**DMI-20**		**Vision-600**			**DMI-20**		**Vision-600**	
				Test	Retest	Test	Retest		Test	Retest	Test	Retest
150 − 20%	**150**	20%	**30**	31	30	33	30	**120**	124	127	118	119
150 − 40%	**150**	40%	**60**	61	59	61	61	**90**	98	99	87	81
150 − 60%	**150**	60%	**90**	91	94	94	85	**60**	63	61	60	58
150 − 80%	**150**	80%	**120**	119	120	126	125	**30**	32	31	31	26
200 − 20%	**200**	20%	**40**	41	43	39	42	**160**	171	168	173	158
200 − 40%	**200**	40%	**80**	80	80	100	81	**120**	132	134	122	119
200 − 60%	**200**	60%	**120**	126	131	125	122	**80**	83	80	86	84
200 − 80%	**200**	80%	**160**	163	162	166	164	**40**	41	42	39	31
250 − 20%	**250**	20%	**50**	49	47	51	50	**200**	200	203	222	211
250 − 40%	**250**	40%	**100**	102	99	101	100	**150**	162	166	182	163
250 − 60%	**250**	60%	**150**	153	160	174	146	**100**	103	100	104	114
250 − 80%	**250**	80%	**200**	200	204	222	218	**50**	52	53	56	43

All measurements were repeated twice within two weeks to one month on each PET/CT system, in order to ensure test-retest reproducibility of the flow values, without changing the phantom set-up. All measurements were performed in the same order. Before each measurement session, the peristaltic pump was calibrated using *Qpump* 200 ml/min. The [^15^O]H_2_O bolus was automatically administered into the flow phantom circulation via the [^15^O]H_2_O RadioWaterGenerator (RWG, Hidex Oy, Turku, Finland) system. Both the DMI-20 and Vision-600 system have their individual RWG systems installed next to the PET/CT system gantry.

Both RWGs are daily cross-calibrated with the radionuclide calibrator for [^15^O]H_2_O. In the calibration procedure, the detected counts are time-corrected to the reference point, i.e., the time-point when the peak reaches the end of the infusion line. Based on the corrected counts, decay time, and radionuclide calibrator data, the software generates a calibration coefficient for the detector. Thereafter, the injected activity in units of [MBq] is calculated from the count-rate curve based on the calibration factor. The manufacturer guarantees 15% accuracy within the requested activity production level on the system. The requested activity for each measurement was 500 MBq on both systems.

All injected activities with relative differences between test- and retest measurements are given in Table 3. To investigate the reproducibility of the activity administration between the measurements and the system, the bolus curves were extracted from both injector systems. Overall, due to using two different injector systems, the injected activities for DMI-20 were generally lower than 500 MBq, whereas the activities were higher than 500 MBq for Vision-600, although all the measurements were within the manufacturer specified 15% limit.

**Table 3:** Injected activities for all measurements. The manufacturer guarantees 15% accuracy to 500 MBq.

	DMI-20			Vision-600		
Measurement	Test	Retest	Difference	Test	Retest	Difference
	[MBq]	[MBq]	%	[MBq]	[MBq]	%
150 − 20	496	479	-3.43	482	506	4.98
150 − 40	478	487	1.88	476	507	6.51
150 − 60	482	448	-7.05	513	509	-0.78
150 − 80	444	431	-2.93	517	530	2.51
200 − 20	474	450	-5.06	548	529	-3.47
200 − 40	459	441	-3.92	510	513	0.59
200 − 60	507	480	-5.33	521	536	2.88
200 − 80	514	497	-3.31	527	527	0.00
250 − 20	483	486	0.62	543	506	-6.8
250 − 40	515	472	-8.35	539	544	0.9
250 − 60	492	510	3.66	556	520	-6.47
250 − 80	511	483	-5.48	529	532	0.57

### PET/CT acquisition

PET acquisition and image reconstruction was conducted in dynamic acquisition mode and followed the clinical MPI acquisition protocol used at our institute (Turku PET Centre) [22]. The acquisition duration was divided into 24 time frames, 14 × 5 s, 3 × 10 s, 3 × 20 s, and 4 × 30 s. Prior to each PET acquisition, a measurement-specific CT-based attenuation correction (CTAC) was acquired. The values used in the CT acquisitions were: tube voltage of 120 kV, exposure time of 500 ms, pitch factor of 1.375, tube current of approx. 96 mA (test) and approx. 61 mA (retest) on DMI-20, and tube voltage of 100 kV, exposure time of 500 ms, pitch factor of 1.2, and tube current of approx. 30 mA (test), and approx. 30 mA (retest) on Vision-600.

All data was decay corrected for the injected activity at the start of the measurement, which was derived directly from the data from the injector system. All quantitative corrections were applied for the PET data. Image reconstructions followed the clinical protocol used at our institute [22]. The reconstruction parameters are presented in Table 4.

**Table 4:** Reconstruction parameters used in this study

Reconstruction parameters	DMI-20	Vision-600
**Algorithm**	OSEM	OP-OSEM
**TOF**	TOF	TOF
**PSF**	PSF	PSF
**Iterations**	16	8
**Subsets**	5	5
**Matrix size**	192	220
**Gaussian post-filter [mm]**	5	6
**FOV [cm]**	35	35

### Image analysis

All PET images were analyzed using the QuantifyDCE software (Shelley Medical Imaging Technologies, Ontario, Canada), which is specifically developed for the analysis of the flow phantom data. The details of the image analysis and kinetic modelling are presented in Gabrani-Juma et al. [10]. In short, the software requires the user to define the input chamber and exchange cylinder volumes-of-interests (VOIs) from which the software automatically extracts the input and tissue TACs. Thereafter, the software automatically applies a two-compartmental kinetic model for the TACs, which outputs *Qin*, *Qout*, *delay*, and *ISF* values. The image analysis was conducted similarly for all data sets by a single operator, and ROI locations were fixed for each measurement session.

### Data analysis

For quality control, the flow meter readings from *Qcyl* and *Qtube* were extracted for all measurements and compared in terms of relative difference to the expected value from *Qpump*. We report the relative differences for the sum *Qtube* + *Qcyl* in comparison to expected *Qpump* for all measurements.

The comparison of the bolus curves and TACs was conducted visually as well as quantitatively by evaluation of the areas-under-the-curves (AUCs), whereas the modelled flow values were compared quantitatively and statistically.

The extracted bolus activity curves from the [^15^O]H_2_O generator were inspected visually for each measurement to investigate their reproducibility. Bolus AUCs were computed from each bolus curve to investigate their contribution to the measured input TAC as well as the tissue TAC from the phantom. Both input and tissue TACs with the modelled TACs were inspected visually to assess the reproducibility between the systems.

Thereafter, comparison for *Qin* and *Qout* values was made between all test and retest measurements for both PET/CT systems. The absolute error of *Qin* and *Qout* with respect to *Qref* was calculated as

1 $$Flow\,value\,error\, = \,\left| {\frac{{Flow\,value\, - \,Qref}}{{Qref}} * 100\% } \right|,$$

and is reported in percentage (%) units.

The relative error of modelled flow values was calculated thereafter using the following equation:

2 $$Repeatability\,error\, = \left| {\frac{{Flow\,value\,\left( {retest} \right) - Flow\,value\,\left( {test} \right)}}{{Flow\,value\,\left( {test} \right)}} * 100\,\% } \right|,$$

and is also reported in percentage (%) units. Thereafter, correlation and agreement between *Qin* and *Qout* values was compared on both systems using correlation and Bland-Altman plots.

## Results

**Fig. 1:**
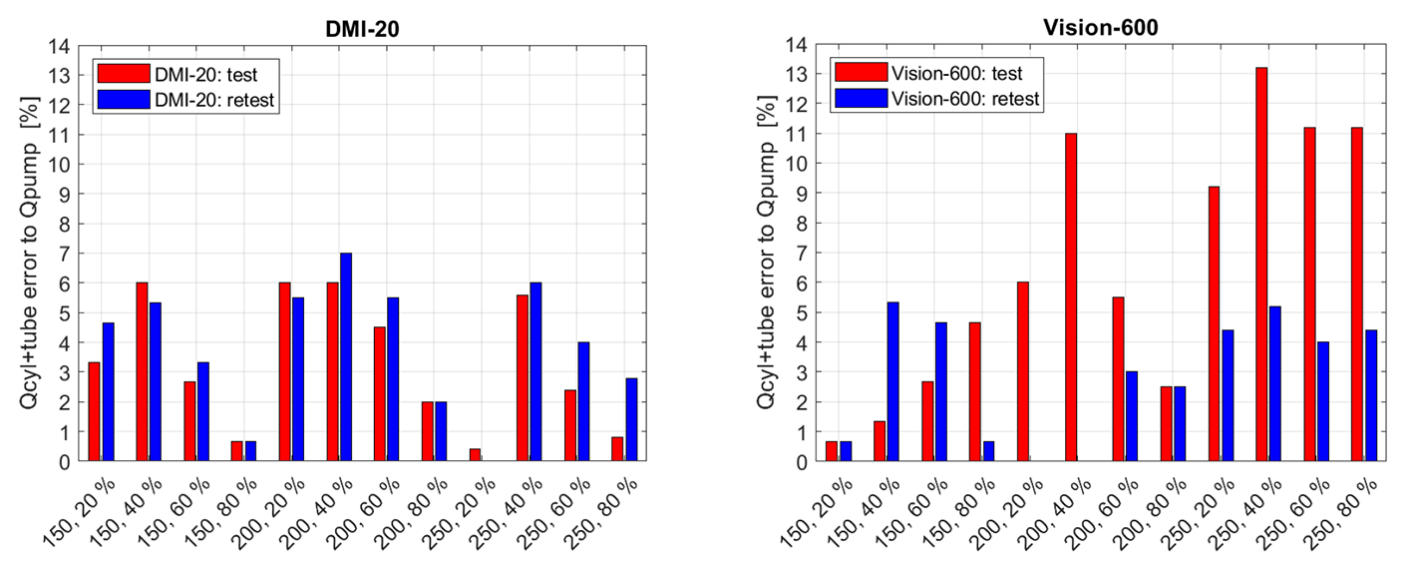
Qcyl + Qtube relative errors with respect to Qpump presented for all measurements on DMI-20 (left) and Vision-600 (right)

Figure 1 shows the differences of the flow meter recordings from the sum of the *Qcyl* and *Qtube* values with respect to the set *Qpump* values on the measurements from DMI-20 and Vision-600. The measurements performed on DMI-20 showed similar difference ranges between test and retest measurement for different flow settings, whereas the measurements performed on Vision-600 produced larger differences on test measurements. In all flow phantom measurements, the flow meter readings showed smaller than 15% relative difference. Overall, the magnitude of the errors was similar for DMI-20 test and retest measurements as well as retest measurements on Vision-600. On Vision-600 200 − 40%, 250 − 40%, 250 − 60%, and 250 − 80% test measurements the relative differences were larger than 10%.

**Fig. 2:**
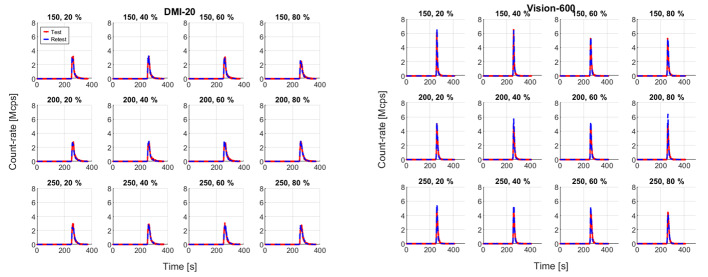
[^15^O]H_2_O bolus curves extracted for each measurement on the RWG system installed on DMI-20 (left) and Vision-600 (right)

**Fig. 3:**
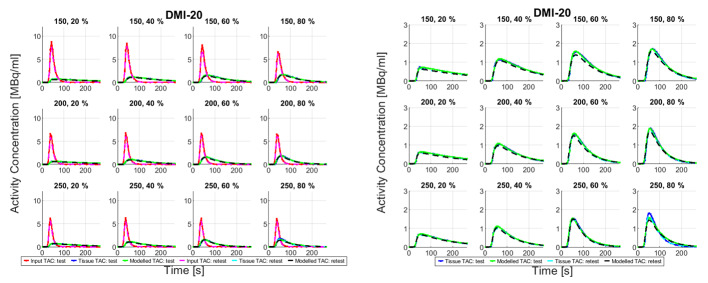
Input, tissue, and modelled time-activity curves (left), and zoomed tissue and modelled TACs (right) recorded on Vision-600 from all measurements

Figure 2 shows the bolus curves extracted from test and retest measurements on both RWG dispenser systems installed on the DMI-20 and Vision-600 PET/CT systems. The RWG installed on the Vision-600 system produced higher bolus peaks compared to the RWG installed on the DMI-20 system. Subsequently, the measurements performed on Vision showed higher injected activities (Table 3) and higher peaks for the input TACs (Fig. 3).

**Fig. 4:**
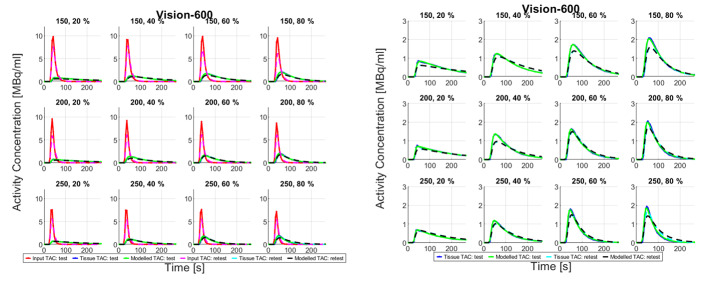
Input, tissue, and modelled time-activity curves (left), and zoomed tissue and modelled TACs (right) recorded on DMI-20 from all measurements

Figures 4 and 3 show TACs from all measurements recorded on both PET/CT systems. There are amplitude differences in input and tissue TACs between the DMI-20 test and retest measurements. On Vision-600 the amplitude differences in input TACs between test and retest measurements are smaller than on DMI-20 but the tissue TACs show differences in shapes between test and retest measurements. The tissue TACs clearly intersect in measurements 150 − 20%, 150 − 60%, 200 − 40%, 250 − 20%, 250 − 60%, and 250 − 80%. The peak amplitudes of all TACs on the measurements on Vision-600 are higher compared to the measurements on DMI-20.

Table 5 shows *Qin* and *Qout* flow values, as well as their flow value errors with respect to *Qref* from Eq. (1) for test and retest measurements. Overall, most of the flow value errors fall below 10% on both test and retest measurements performed on DMI-20. However, the Vision-600 test and retest measurements show higher flow value errors especially with *Qout* compared to DMI-20, several being larger than 10%.

**Table 5:** Modelled Qin and Qout values as well as their absolute relative errors with respect to Qref from all measurements on both DMI-20 and Vision-600

Measurement	DMI-20		Vision-600		DMI-20		Vision-600		DMI-20		Vision-600		DMI-20		Vision-600	
	Qin		Qin		Qout		Qout		Qin error		Qin error		Qout error		Qout error	
	Test	Retest	Test	Retest	Test	Retest	Test	Retest	Test	Retest	Test	Retest	Test	Retest	Test	Retest
150 − 20	40	37	49	37	38	37	62	46	19	23	5	23	22	24	22	4
150 − 40	72	72	81	79	63	64	90	89	8	6	4	1	19	15	15	14
150 − 60	111	111	112	99	106	107	122	111	2	1	1	3	2	4	10	9
150 − 80	146	145	140	134	138	140	153	148	6	5	4	7	0	1	5	2
200 − 20	49	50	48	47	43	47	55	54	15	17	15	21	27	22	3	9
200 − 40	99	99	112	94	94	93	127	106	2	2	4	4	3	4	8	8
200 − 60	159	156	139	135	156	154	162	155	9	3	3	4	7	2	12	10
200 − 80	188	181	186	164	187	185	208	177	2	5	5	15	3	3	6	8
250 − 20	69	68	61	55	64	62	76	60	4	6	10	17	3	3	12	10
250 − 40	118	121	120	118	118	118	134	135	1	4	1	0	1	2	13	15
250 − 60	182	197	193	181	181	205	224	214	2	5	5	6	1	9	10	26
250 − 80	179	179	231	172	176	174	270	188	22	23	8	30	23	26	7	24

**Fig. 5:**
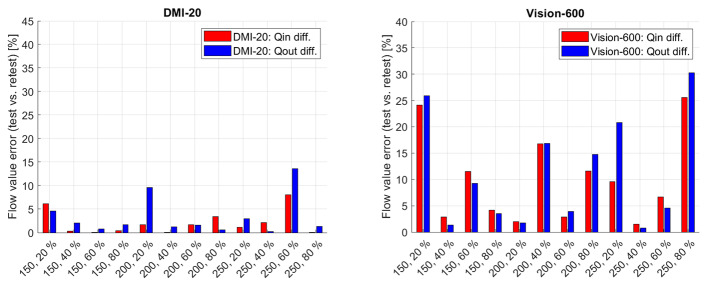
Qin and Qout differences between test and retest measurements on DMI-20 (left) and Vision-600 (right)

Figure 5 shows repeatability errors of *Qin* and *Qout* between test and retest measurements (Eq. 2) on the measurements on DMI-20 and Vision-600. All DMI-20 measurements fall below 15% repeatability errors whereas Vision-600 shows larger than 15% errors on four measurements (150 − 20%, 200 − 40%, 250 − 20%, and 250 − 80%). The mean ± standard deviation of errors for DMI-20 *Qin* and *Qout* are 2.1% ± 2.6% and 3.3% ± 4.1%. The corresponding numbers for Vision-600 *Qin* and *Qout* are 10% ± 8.4% and 11% ± 10%, respectively.

**Fig. 6:**
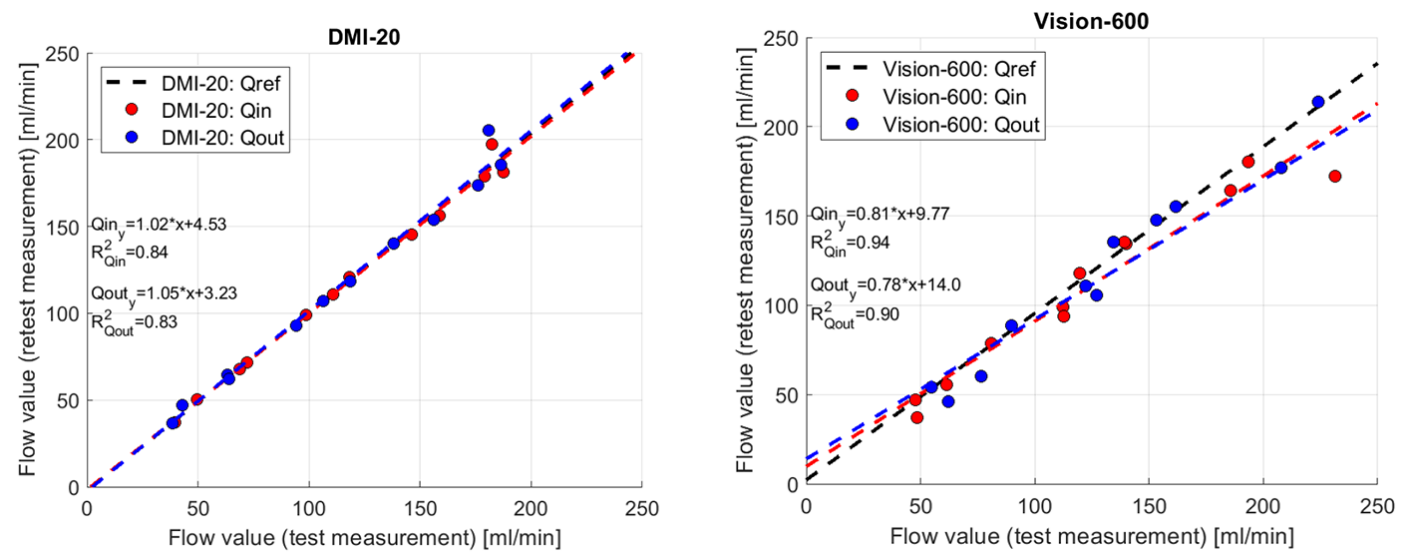
Correlation of DMI-20 (left) and Vision-600 (right) test and retest measurements presented with the reference flow line

Figure 6 shows that *Qin* and *Qout* values are close to the reference flow values (*Qref*) on DMI-20 and show resemblance between test and retest measurements. In addition, the linear polynomial fits are close to the reference line for both DMI-20 *Qin* and *Qout*. In comparison, on the measurements performed on Vision-600 both *Qin* and *Qout* values diverge from the reference line (Fig. 6) but the linear polynomial fit is similar for both Qin and Qout.

**Fig. 7:**
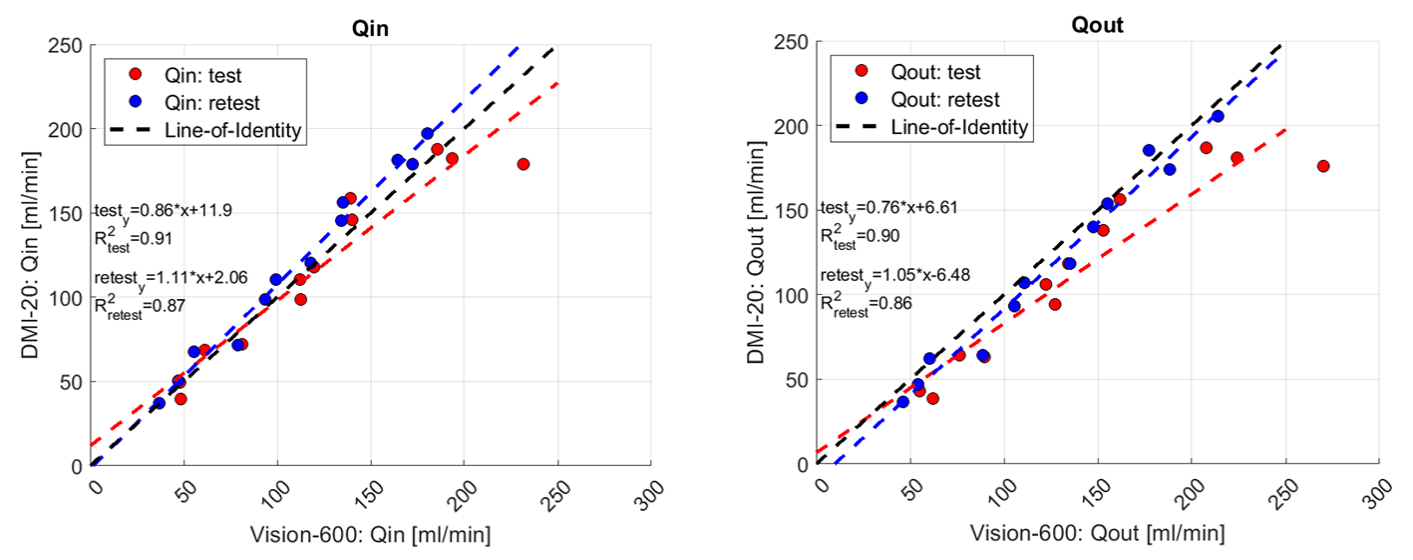
Qin (left) and Qout (right) values extracted from test and retest measurements for DMI-20 and Vision-600. The fitted lines show the correlation between DMI-20 and Vision-600 separated for the test- and retest measurements and line-of-identity the ideal correlation between DMI-20 and Vision-600

Figure 7 describes the correlation of *Qin* and *Qout* flow values between DMI-20 and Vision-600 test and retest measurements. For *Qout* values the retest measurements are close to the line-of-identity and *Qin* test and retest measurements have similar deviation from the line-of-identity. The deviation between DMI-20 and Vision-600 is higher with larger flow rates in test and retest measurements for *Qin* and for test measurements for *Qout*.

**Fig. 8:**
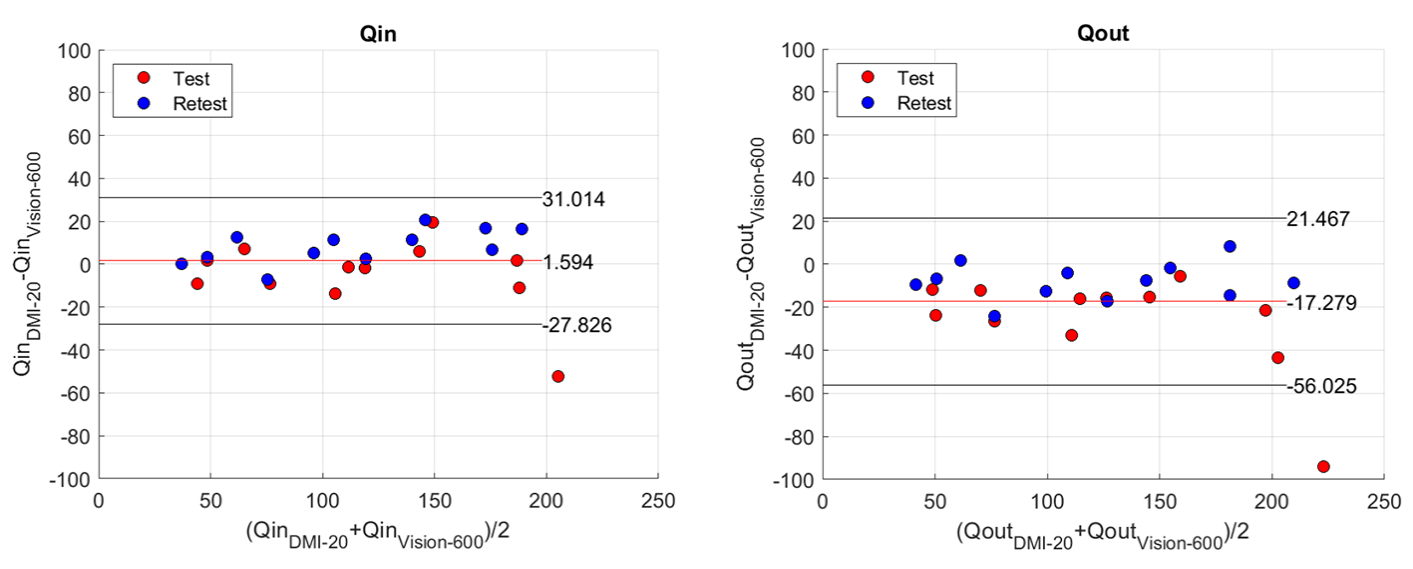
Bland-Altman plots for Qin (left) and Qout (right) value between DMI-20 and Vision-600

The Bland-Altman plots in Fig. 8 show that the *Qout* values vary more compared to Qin between DMI-20 and Vision-600. The lines-of-agreement (LoAs) are larger for *Qout* compared to *Qin* LoAs. Also, the mean differences between DMI-20 and Vision-600 are 1.59 ml/min and −17.3 ml/min for *Qin* and *Qout*. There is one outlier point for the test measurement in both *Qin* and *Qout*. The outlier is measurement 250 − 80% where DMI-20 produces test *Qin* and *Qout* values of 179 ml/min and 176 ml/ min and the corresponding values for Vision-600 are 231 ml/min and 270 ml/min.

## Discussion

This study proposed a preliminary protocol for assessing the technical factors contributing to the accuracy and reproducibility of quantitative flow values in MPI. This protocol could eventually be used for planning harmonization measurements for myocardial perfusion imaging using [^15^O]H_2_O as well as the flow phantom in the future. We evaluated the impact of technical factors on the modelled flow values, *Qin* and *Qout*, extracted from the flow phantom. The accuracy and reproducibility was evaluated between test and retest measurements, two [^15^O]H_2_O injectors as well as two digital PET systems, Discovery MI and Biograph Vision 600. There is no specific reason why the protocol could not be applied to analog PET/CT systems as well.

First, we evaluated how well the measured *Qcyl* and *Qtube* flow values inside the phantom fulfill the ideal pre-assumption that *Qtube + Qcyl* should be equal to *Qpump*. Almost all measurements on both DMI-20 and Vision-600 showed errors between *Qtube + Qcyl* vs. *Qpump*. Especially the test measurements on Vision-600 produced larger *Qtube + Qcyl* differences to *Qpump* when compared to the retest measurement on Vision-600, or test and retest measurement on DMI-20. It is still worth noticing that all *Qtube + Qcyl* vs. *Qpump* differences were below 15% on both systems (Fig. 1). This finding suggests that the flow phantom set-up is a technical factor that plays a role in the measurement accuracy.

Considering in applying the phantom for larger multi-institutional studies, the flow phantom has internal characteristics that may affect the measurement. For example, the pressure variations inside the hoses, the air bubbles within the phantom, the air pressure and humidity of the scanner room, and the phantom flow meter inaccuracies can affect the modelled flow values. In addition, the flow phantom presents only a simplified simulation of myocardial perfusion. In clinical subjects, there will be more variations due to physiological factors, including tracer dispersion and individual reactions to pharmacological stress, in addition to other physiological factors that contribute to the measured MBF. However, with correct procedures implemented the phantom can be used to investigate the technical factors independent of the physiological characteristics.

What is more, the phantom vendor advices performing quality control procedures prior conducting the measurements. One procedure is calibrating the peristaltic pump for a measurement session. We however noticed that the measurements could benefit even more by calibrating the pump whenever we altered *Qpump* or the constriction of *Qcyl* or *Qtube* between tests. Although noting this effect, we decided to use a single pump calibration factor between measurements. What is more, the flow meter calibration applied in the QuantifyDCE software should verify the flow meter readings and therefore a single pump calibration should be sufficient. Given that the errors were smaller than 15% and that these variations would still be accounted in kinetic modelling, we assume the *Qtube + Qcyl* error is not the most significant factor affecting the accuracy of the modelled flow values.

Second, we measured the administered [^15^O]H_2_O activities as well as bolus peaks in all measurements and assessed their performance between test and retest measurements as well as the PET/CT systems. The administered activities were repeatable between test and retest measurements with relative differences lower than 9% on both systems (Table 3). All administered activities were within 15% of the requested 500 MBq and fell within the vendor specifications. When comparing the PET/CT systems, the RWG installed on the Vision-600 produced systematically higher activities as well as bolus peak amplitudes. The variation in bolus peak amplitudes is possibly due to the discrepancies in cross-calibration of the two RWG systems. Therefore, the use of individual RWGs on each PET/CT system might be a technical factor affecting the measurement accuracy, especially between the PET/CT systems used.

Third, we evaluated the accuracy and reproducibility of the modelled flow values. The errors of *Qin* and *Qout* with respect to *Qref* were smaller on DMI-20 compared Vision-600 showing higher accuracy on DMI-20. Also, we could show that on DMI-20 all measurements were highly repeatable as the repeatability errors of *Qin* and *Qout* were below 15% (Fig. 5; Table 5). In comparison, on Vision-600 the repeatability errors were higher for almost all measurements and increased as high as 40% on one measurement (150 − 20%). Moreover, the correlation plots of *Qin* and *Qout* between DMI-20 and Vision-600 showed discrepancies (Fig. 7) with more variability on higher flow rates (Fig. 8). This variability will likely have higher impact on MPI patients during pharmacological stressing due to higher MBF.

The most probable explanation for the reproducibility differences between DMI-20 and Vision-600 is the difference in tissue TACs between the test and retest measurements on Vision-600. After careful inspection we noted that whenever there was a clear shape discrepancy in the tissue TACs or the tissue TACS were intersecting between test and retest measurements, there was a larger test-retest error in Vision 600. On DMI-20 we did not observe such a trend (see Supplementary File 1).

This finding indicates that even though the measurement set-ups between test and retest sessions were as repeatable as possible, there still exists a fundamental source of variability between the two PET/CT systems, which can be attributed to be originating from technical factors. Therefore we strongly advice investigating other factors affecting the flow value modelling accuracy. For example, one possible factor affecting the discrepancy might be the reconstruction differences between Vision-600 and DMI-20. EARL has proposed guidelines in oncological imaging to apply reconstruction parameters that result into equal voxel sizes regardless of the PET system used [4]. In this study, on DMI-20 the voxel sizes were (*x*, *y*, and *z*) 1.82 mm, 1.82 mm, and 2.79, and on Vision-600 1.65 mm, 1.65 mm, and 3. Thus, the final reconstructed image resolution on both systems was similar. We still advise studying this phenomenon in more detail and perform reconstruction harmonization studies between DMI-20 and Vision-600 in order to eliminate this factor from affecting the modelling accuracies.

What is more, one factor that might explain the differences between the systems may be the higher sensitivity of the Vision-600 compared to DMI-20 (13.7 cps/MBq on DMI-20 and 16.4 cps/MBq on Vision-600) as well as smaller spatial resolution of Vision-600 compared to DMI-20 (4.1 mm on DMI-20 and 3.5 mm on Vision-600) (Table 1). Additional factor affecting the differences between DMI-20 and Vision-600 is in the implemented scatter correction algorithm and the difference in its behavior. The scatter correction factors especially in the Vision-600 measurements were seen to vary across time between test and retest measurements, although no such behavior was seen on the DMI-20 (please see Supplementary File 2). Finally, even though we could not show any direct impact between bolus amplitudes and flow value modelling differences, this is a factor that should be minimized between the PET/CT systems used.

In summary, this study has identified the following technical factors affecting the accuracy and reproducibility of modelled flow values between as well as within the PET/CT systems. The factors are (1) the flow phantom set-up, (2) the injected bolus, (3) the RWG producing the injected bolus, (4) the flow-rate, (5) the reconstruction parameters, (6) the sensitivity of the PET/CT systems, and (7) the scatter correction algorithm, as well as any subsequent data corrections. The contribution of these factors should be investigated in more detail.

When comparing the results of this study to the data published from MBF values measured on clinical subjects, the mean values and standard deviations of the repeatability errors between test and retest measurements on Vision-600 (*Qin*: 10% ± 8.4% and *Qout*: 11% ± 10%) or DMI-20 (*Qin*: 2.1% ± 2.6% and *Qout*: 3.3% ± 4.1%) were within a similar or smaller range that is measured in clinical myocardial perfusion studies [23]. For example the table in Klein et al. describes that overall the stress MBF values have repeatability accuracy from 11 to 34%, and for [^15^O]H_2_O the reported values are 27% [18] and 25% [19]. In this regard, the measured values from the phantom study agree well with the results gained from the clinical subjects and brings light on the level of reproducibility achieved without biological factors. As compared to our phantom study, higher variation in patient studies reflect the contribution of differences in biological factors, such as the systemic hemodynamic state, on MBF.

In the future, there is room for validating the protocol for multiple tracers used in MPI. The present protocol was assessed only for [^15^O]H_2_O but also [^82^Rb] and [^13^N]NH_3_ are commonly used in MPI as perfusion tracers. Thus, at least a cross-verification to the present protocol or designing a tracer-specific protocol for these tracers is required. Moreover, modifying the protocol to be used with [^18^F]-labelled tracers would be highly useful, as they are widely available in different centers. Thereafter, preliminary harmonization measures could be evaluated using also ^18^F-tracers, as long as the protocol would have been cross-calibrated with the present [^15^O]H_2_O protocol.

### Summary of the findings

In this study we were able to demonstrate 15% repeatability across all measurements on DMI-20 and on 7/24 measurements on Vision-600. This relatively high repeatability is expected, as in a single-center setting there are several factors that are advantageous for minimizing the bias and variability between the measurements. First, the [^15^O]H_2_O bolus injectors were calibrated to a common reference within the center. Second, the acquisition protocols and the activity delivery were standardized between the measurements. Third, both PET systems were cross-calibrated to the common reference within the center, their acquisition durations as well as reconstruction frame times were the same, with relatively similar settings in the reconstruction parameters.

These experiments confirmed that the proposed imaging protocol with several flow values as well as imaging parameters including reconstructions can be applied between different injector and PET/CT systems to provide an understanding for technical accuracy and reproducibility of the quantitative flow values between test-retest measurements. However, after all there still resumes an underlying question: where do the higher errors on Vision-600 repeatability origin from as on DMI-20 such errors were not visible? This calls for further attention to assess and minimize the contribution of different factors between PET/CT systems.

A future multi-center study would be essential to provide upper and lower limits for the *Qin* and *Qout* values for calibration purposes and would give specific and more detailed information about the different factors contributing to the variations of MBF values between centers and PET/CT systems. Eventually these findings would provide information for establishing similar quality control measures that EARL recommends for oncological PET imaging. Before applying this protocol for multi-center setting, a careful investigation for standardizing the bolus delivery between different sites and dispenser systems needs to be conducted first. Ensuring an even more consistent tracer administration profile should improve test-retest repeatability as well as system-to-system reproducibility further [23]. However, in multi-center settings there will be several more technical factors affecting the measurements, all of which should be investigated separately.

## Conclusions

A preliminary protocol for measuring the accuracy and reproducibility of flow values in [^15^O]H_2_O MPI between digital PET/CT systems was assessed. The test-retest reproducibility falls below 15% in majority of the measurements conducted between two individual injector systems and two digital PET/CT systems. This study highlights the importance of implementing a standardized bolus injection and delivery protocol, which should be carefully investigated in a multi-center setting. The study indicated that there still remain a number of technical factors which could be investigated further, to further minimize their effect to accuracy and reproducibility of flow values within and between PET/CT systems.

### Electronic supplementary material

Below is the link to the electronic supplementary material.

Supplementary Material 1

Supplementary Material 2

## Data Availability

The datasets used and/or analysed during the current study are available from the corresponding author on reasonable request.
