# Supplementary material for: Preliminary protocol for measuring the reproducibility and accuracy of flow values on digital PET/CT systems in [15O]H2O myocardial perfusion imaging using a flow phantom
Source: EJNMMI Phys. 2024 Jul 1;11:54. doi: 10.1186/s40658-024-00654-y (PMC11217201; doi:10.1186/s40658-024-00654-y)
Supplement: Supplementary file 1 — Supplementary Material 1 [file 40658_2024_654_MOESM1_ESM.docx]

Suppelementary File 1

After careful inspection we noted that whenever there was a clear shape discrepancy in the tissue TACs or the tissue TACS were intersecting between test and retest measurements, there was a larger test-retest error on Vision 600. On DMI-20 we did not observe such a trend.

Example figure of the Vision 200-40 % test-retest tissue TACs shows that there is a clear intersect between the measurements and also there was a large difference (17 %) in flow values between test and retest on both Qin and Qout. In comparison, Vision-600 200-60 % test-retest tissue TACs show only an amplitude difference and therefore the differences in flow values was not prominent (3 % for Qin and 4 % for Qout) as the tissue TACs were not intersecting.


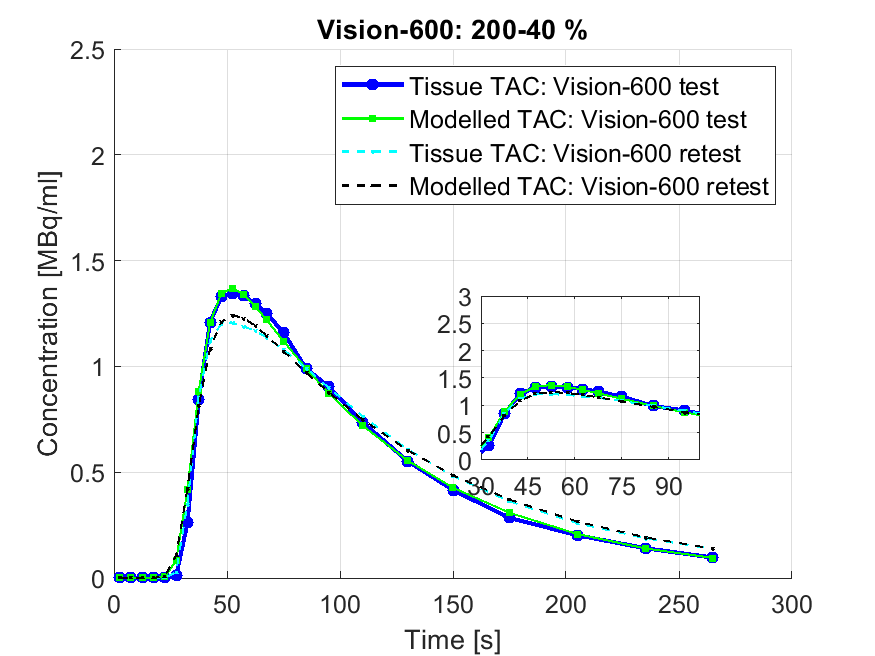

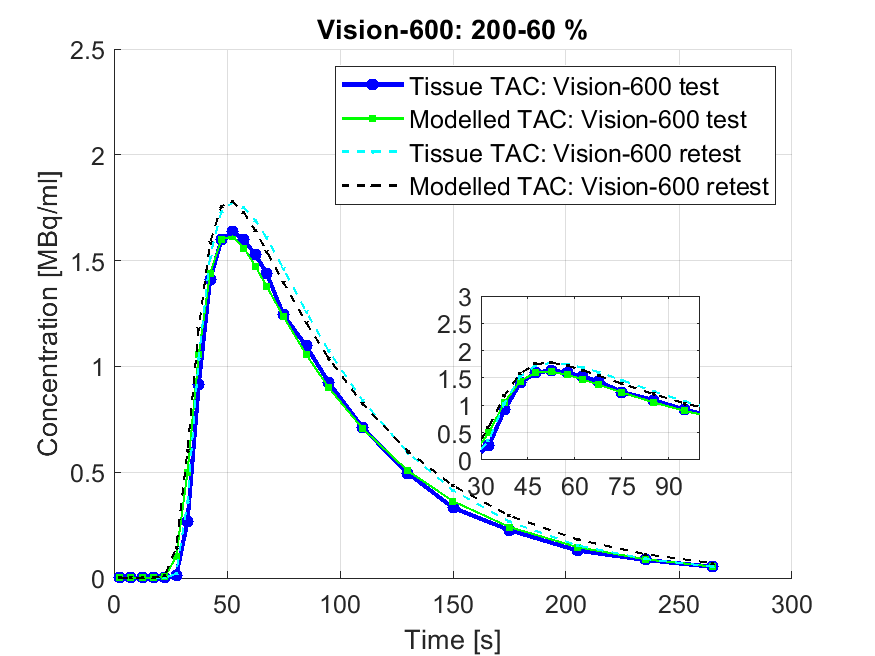


In the measurements on DMI-20 there the tissue TACs were not intersecting. These inspections showed us that a factor that affects the flow value accuracy clearly is the tissue TAC behavior between measurements. As the tissue TACs are different between DMI-20 and Vision-600 that creates a clear difference in flow values between the systems.
