# Supplementary material for: Preliminary protocol for measuring the reproducibility and accuracy of flow values on digital PET/CT systems in [15O]H2O myocardial perfusion imaging using a flow phantom
Source: EJNMMI Phys. 2024 Jul 1;11:54. doi: 10.1186/s40658-024-00654-y (PMC11217201; doi:10.1186/s40658-024-00654-y)
Supplement: Supplementary file 2 — Supplementary Material 2 [file 40658_2024_654_MOESM2_ESM.docx]

Appendix 1

Example of the scatter fraction factors (SF) from the measurement 200-40 % on both Vision-600 and DMI-20 in Figure 1.


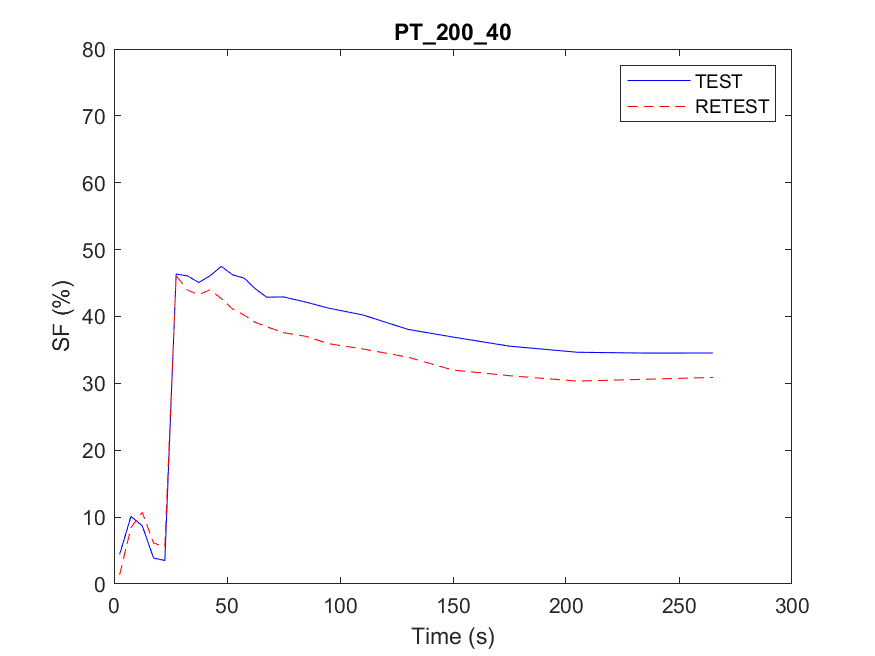

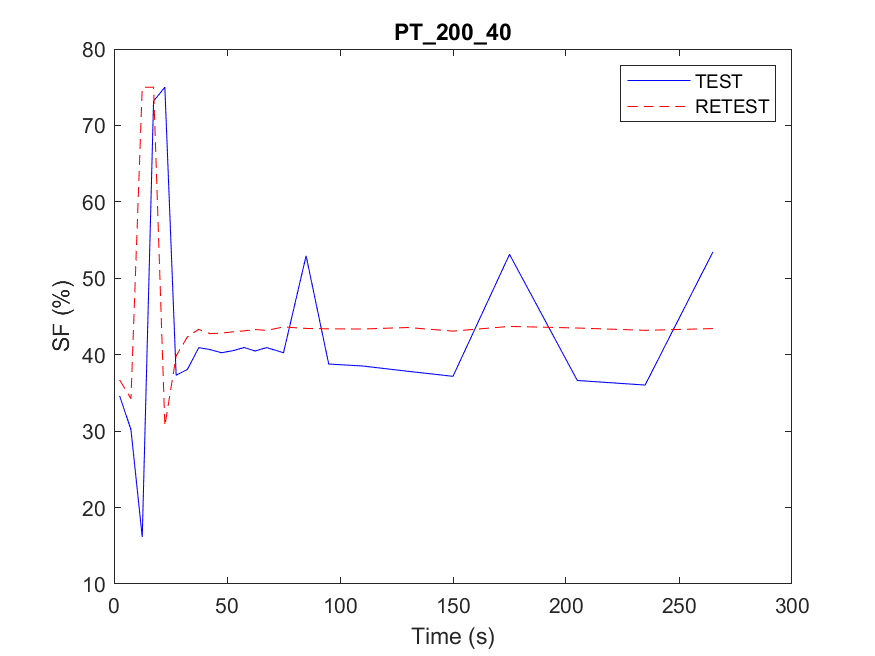


Figure 1. DMI-20 (left) shows no large variation in SFs during the data acquisition whereas Vision-600 (right) shows variation in SFs over time.
